# Unfolding the Network of Peer Grades: A Latent Variable Approach

**DOI:** 10.1017/psy.2025.10021

**Published:** 2025-06-16

**Authors:** Giuseppe Mignemi, Yunxiao Chen, Irini Moustaki

**Affiliations:** 1 Department of Decision Sciences, Bocconi University, Milan, Italy; 2 Department of Statistics, London School of Economics and Political Science, London, UK

**Keywords:** Bayesian modeling, cross-classified model, peer grading, rater model

## Abstract

Peer grading is an educational system in which students assess each other’s work. It is commonly applied under Massive Open Online Course (MOOC) and offline classroom settings. With this system, instructors receive a reduced grading workload, and students enhance their understanding of course materials by grading others’ work. Peer grading data have a complex dependence structure, for which all the peer grades may be dependent. This complex dependence structure is due to a network structure of peer grading, where each student can be viewed as a vertex of the network, and each peer grade serves as an edge connecting one student as a grader to another student as an examinee. This article introduces a latent variable model framework for analyzing peer grading data and develops a fully Bayesian procedure for its statistical inference. This framework has several advantages. First, when aggregating multiple peer grades, the average score and other simple summary statistics fail to account for grader effects and, thus, can be biased. The proposed approach produces more accurate model parameter estimates and, therefore, more accurate aggregated grades by modeling the heterogeneous grading behavior with latent variables. Second, the proposed method provides a way to assess each student’s performance as a grader, which may be used to identify a pool of reliable graders or generate feedback to help students improve their grading. Third, our model may further provide insights into the peer grading system by answering questions such as whether a student who performs better in coursework also tends to be a more reliable grader. Finally, thanks to the Bayesian approach, uncertainty quantification is straightforward when inferring the student-specific latent variables as well as the structural parameters of the model. The proposed method is applied to two real-world datasets.

## 1 Introduction

**Figure 1:**
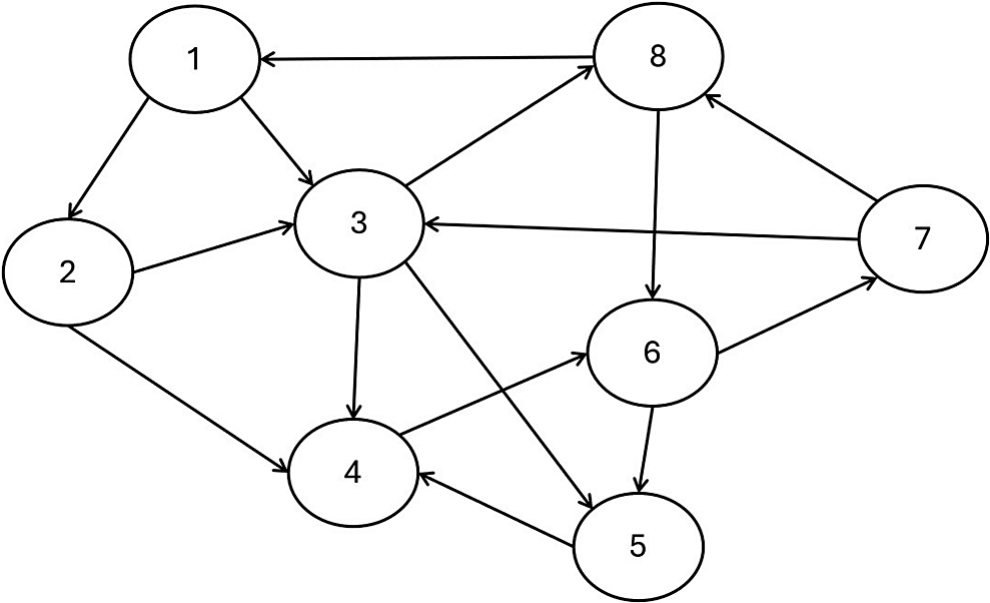
Network diagram representing the network structure of peer grading data. *Note*: Each circle is a vertex of the network and represents a student. The arrows are the peer grades, which serve as edges connecting two students; their direction indicates whether the student receives or gives the grade.

Peer grading, also known as peer assessment, is a system of formative assessment in education whereby students assess and give feedback on one another’s work. It substantially reduces teachers’ burden for grading and improves students’ understanding of the subject and critical thinking (Panadero & Alqassab, 2019; Yin et al., 2022). Consequently, it is widely used in many educational settings, including massive open online courses (MOOCs; Gamage et al., 2021), large university courses (Double et al., 2020), and small classroom settings (Sanchez et al., 2017). In a peer grading system, each student’s work is assigned, often randomly, among several other students who act as graders or raters. Due to the design of this system, peer grading data have a different structure from traditional rating data, which also consists of students’ grades from graders. For traditional rating data, the students whose work is evaluated cannot serve as graders, which leads to a relatively simple data structure. On the other hand, peer grading data have a network structure where all the peer grades may be dependent. Each student can be viewed as a network vertex, and each peer grade serves as an edge connecting two students—a grader and an examinee (see Figure 1 for a visual illustration of such a network structure).

A simple peer grading system aggregates the peer grades using a straightforward method like the mean or median to derive a final grade for each student’s work (Reily et al., 2009; Sajjadi et al., 2016). This conventional method does not consider the heterogeneity among the graders. Some graders may exhibit systematic biases and tend to assign higher or lower grades than their peers when assessing the same work. Graders may also exhibit varying levels of reliability; while some maintain consistent grading standards, others may give erratic grades that lack a consistent standard. Furthermore, when the data involve multiple formative assessments for each student, a more accurate grade may be derived by borrowing information across assessments. Finally, monitoring how students perform as graders is often helpful, as it provides an opportunity to reward the best-performing graders and offer feedback to help those who need improvement. Different methods have been developed to mitigate grader bias and improve peer assessment reliability (see Alqassab et al., 2023 for a review). Depending on whether instructors’ scores are needed in method training, they can be classified as supervised and unsupervised learning methods. Supervised learning methods utilize instructors’ scores to train a function that maps multiple peer grades to an aggregated grade that mimics the instructor’s score (Namanloo et al., 2022; Xiao et al., 2020). For instance, Namanloo et al. (2022) proposed a graph convolutional network method that uses peer grades and behaviors of peers to predict the respective instructors’ scores.

On the other hand, unsupervised learning methods try to find an aggregation rule based only on peer grades without access to instructors’ scores. Unsupervised learning is typically performed by employing latent-variable-based measurement models (e.g., Han, 2018; Piech et al., 2013; Xu et al., 2021), which are closely related to models for traditional rating data in which each individual is either a student or a grader. As explained in the sequel, they make an independence assumption that is also adopted in the latent variable models for traditional rating data. However, as peer grading data have a complex network structure, this independence assumption is likely oversimplified, leading to suboptimal performance.

Many latent variable models have been proposed for traditional rating data, including the facet model (Linacre, 1989) and its extensions (Uto & Ueno, 2020; Uto, 2021), the hierarchical rater models (Casabianca et al., 2016; DeCarlo et al., 2011; Molenaar et al., 2021; Nieto & Casabianca, 2019; Patz et al., 2002), the rater bundle model (Wilson & Hoskens, 2001), and the generalized rater model (Wang et al., 2014). These models introduce rater-specific parameters to model the rater effects in the data. With many raters, these rater-specific parameters are treated as random effects (i.e., latent variables) and further assumed to be independent of the examinee-specific latent variables used to model examinee performance. These assumptions are also made in the existing latent variable models for peer grading data (Han, 2018; Piech et al., 2013; Xu et al., 2021). However, we note that the assumption about the independence between the rater-specific latent variables and examinee-specific latent variables does not hold for peer grading data, as the same students are both examinees and raters, and the characteristics of the same student as a rater and those as an examinee are naturally correlated. Ignoring such dependence can result in model misspecification and substantial information loss. To the best of our knowledge, no rater model in the literature accounts for such a dependence structure.

We fill this gap by proposing an unsupervised latent variable model for peer grading data. The proposed model jointly analyzes peer grades for multiple assessments and produces more accurate aggregated grades. It models the student effects with correlated latent variables that capture a student’s characteristics as an examinee and a grader, respectively. Unlike the existing latent variable models for peer grading data, the proposed model captures the dependence in data brought by the network structure of peer grades and the dual roles of each student as an examinee and a rater.

Due to the complex dependence structure under the proposed model, its marginal likelihood involves a very high-dimensional integral with respect to all the student-specific latent variables that can hardly be simplified. Thus, solving the maximum likelihood estimator is computationally infeasible, and consequently, frequentist inference based on the marginal likelihood is a challenge. We develop a fully Bayesian approach for drawing statistical inferences to overcome the computational challenge. With this approach, uncertainty quantification is straightforward when inferring the student-specific latent variables as well as the structural parameters of the model. However, its computation is still non-trivial due to the presence of a large number of latent variables and a complex network structure. To solve this, we use a No-U-Turn Hamiltonian Monte Carlo (HMC) sampler (Hoffman & Gelman, 2014), which produces efficient approximate samples from the posterior distribution.

Besides the traditional rater models, the proposed framework is closely related to cross-classified random effects models (Goldstein, 1994; Rasbash & Goldstein, 1994; Raudenbush, 1993), an extension of standard multilevel models for non-hierarchical data that have cross-classified structures. These models have received wide applications for evaluating measurement reliability, including in generalizability theory (Brennan, 2001, 2010). Our data involve three crossed factors—the examinees, the graders, and the assessments—and the proposed model decomposes each peer grade based on these three factors. However, our model allows the latent variables (i.e., random effects) associated with the crossed factors (examinees and raters) to be correlated to account for the special design of peer grading. In contrast, a standard cross-classified random effects model assumes the random effects associated with different crossed factors to be independent. Introducing such dependence among the latent variables substantially increases the complexity of the model and its inferences. Our model also has close connections with several latent variable models concerning dyadic data, including social relations models (e.g., Kenny & La Voie, 1984; Nestler, 2016; Nestler et al., 2017; Nestler et al., 2020; Warner et al., 1979) and the dyadic item response theory (IRT) model (Gin et al., 2020), where the dyadic IRT model extends the social relations models by incorporating an IRT measurement model. Peer grading data can be viewed as a special type of dyadic data, where each dyad involves an examinee and a grader, and the dyads are formed by random assignment. However, our model differs substantially from the existing social relations models in how latent variables are modeled and interpreted. The traditional social relations models focus on inferring the causes and consequences of interpersonal perceptions and judgments. In contrast, the current analysis focuses on measuring latent traits concerned with applying peer grading (e.g., examinee performance and rater reliability). As a result, the existing social relations models are unsuitable for the current application.

The rest of the article is organized as follows. Section 2 proposes a latent variable model framework for peer grading data, within which specific models are discussed. Two real data applications are given in Section 3. Section 4 discusses advantages, limitations, and future directions. The appendix includes extensions of the proposed model, technical details, and additional simulated examples. The Online Supplementary Material include further results from simulation studies and real data analysis.

## 2 Proposed model

### 2.1 Problem setup

Consider *N* students who receive *T* assessments. Each student *i*’s work on assessment *t* is randomly assigned to a small subset of other students to grade their work. We denote this subset as 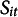 , which is a subset of 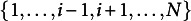 . Each grader 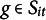 gives this work a grade 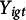 , following certain scoring rubrics. For simplicity, we consider the case when 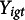 is continuous. It is common, but not required, for the number of graders 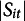 to be the same for all students and assessments. An aggregated score is then computed as a measure of student *i*’s performance on the *t*th assessment, often by taking the mean or the median of the peer grades 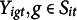 . We note that a simple aggregation rule, such as the mean and the median of the peer grades, fails to account for the grader effect and, thus, may not be accurate enough.

### 2.2 Proposed model

#### Modeling peer grade 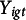


2.2.1

We assume the following decomposition for the peer grade 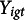 : 
(1)

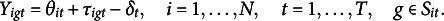

Here, 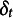 captures the difficulty level of assessment *t*. A larger value of 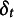 corresponds to a more difficult assessment. In addition, 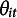 represents student *i*’s true score for assessment *t*, and 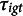 is an error attributed to the grader. We assume 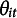 , 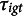 and 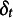 to be independent.

#### Modeling true score 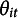


2.2.2

For each student *i*, we assume that their true scores for different assessments 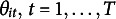 , are independent and identically distributed (i.i.d.), following a normal distribution 
(2)

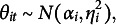

where the mean and variance are student-specific latent variables. The latent variable 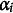 captures the student’s average performance over the assessments, and the latent variable 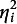 measures their performance consistency (i.e., the extent to which students’ proficiency varies across assessments). This model assumes the true scores fluctuate randomly around the average score 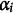 without a trend. This assumption can be relaxed if we are interested in assessing students’ growth over time (see Appendix B to relax this assumption).

#### Modeling grader effect 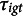


2.2.3

Each student *g* grades multiple assessments from multiple students. We let 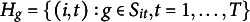 be all the work student *g* grades. For each student *g*, we assume that 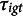 , for all 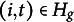 , are i.i.d., following a normal distribution 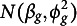 , where the mean and variance are student-specific latent variables. The latent variable 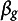 may be interpreted as the bias of student *g* as a grader. For two students *g* and 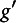 satisfying 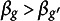 , student *g* will give a higher grade on average than student 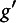 when grading the same work. We say grader *g* is unbiased when 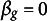 . Moreover, the latent variable 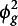 measures the grader’s reliability. A smaller value of 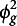 implies that the grader provides consistent grades to similar quality assessments, while a larger value suggests the opposite. In other words, when grading multiple pieces of work with the same true score and assessment difficulty (so that, ideally, they should receive the same grade), a grader with a small 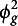 tends to give similar grades, and thus, the grades are more reliable. In contrast, a grader with a large 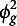 tends to give noisy grades that lack consistency. We remark that the grader effects 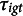 , 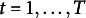 , are assumed to be i.i.d. in the current setting, which means the grading quality remains the same across assignments.

#### 2.2.4 Joint modeling of student-specific latent variables

The model specification above introduces four latent variables, namely 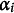 , 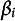 , 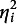 , and 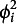 , for each student *i*. These variables allow us to account for the relationship between a student’s performance data and grading data as an examinee and a grader. By allowing for dependence between these variables, we can share information and make more informed evaluations of their performance. We assume that 
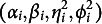
 , where 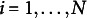 are i.i.d.; we also assume that 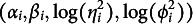 follows a multivariate normal distribution 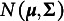, where 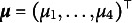 and 
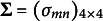
. To ensure parameter identifiability, we set 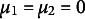 so that the average score of each assessment (averaged across students and graders) is completely captured by the difficulty parameter 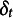 . There are no constraints on 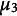 and 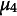 .

#### 2.2.5 Remarks

**Figure 2:**
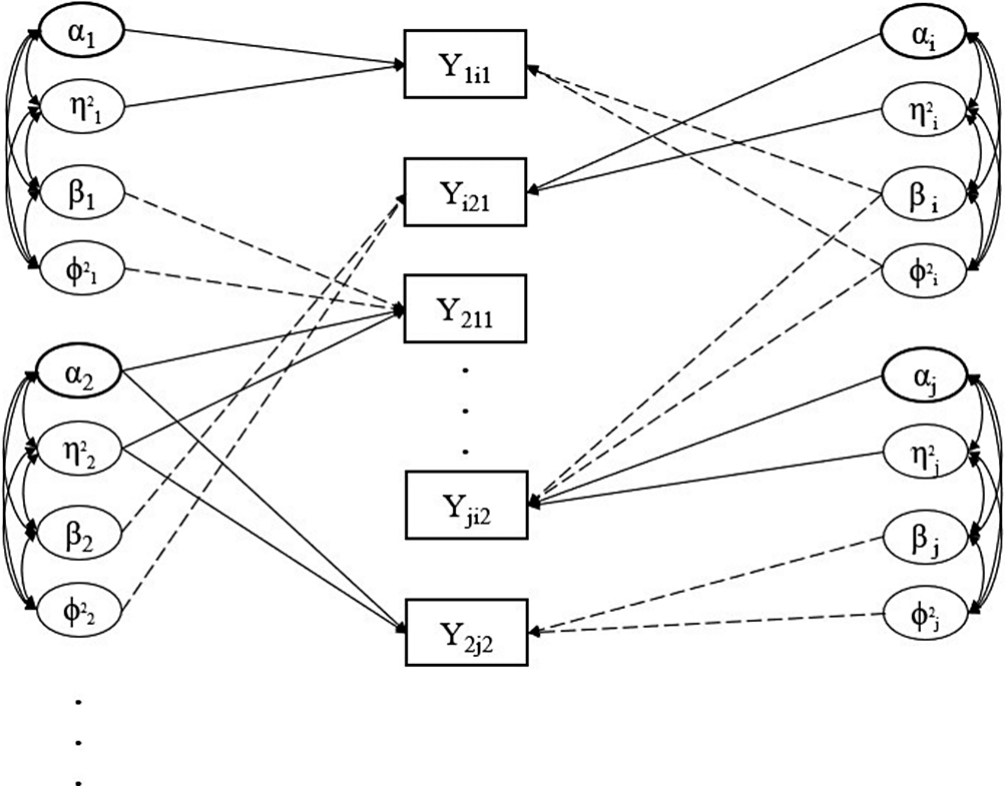
Path diagram representing the network structure of peer grading data. *Note*: The latent variables of four independent students are represented as an example. Students’ grades, reported in the squared box, refer to two assessments, as the subscripts indicated. The curve double-arrows stand for correlation; the straight (solid and dotted) lines represent the effect of the respective latent variable. For the sake of readability, we prefer to adopt the solid lines for the effect of variables referring to the role of the examinee (i.e., 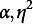 ), whereas the dotted lines refer to the effect of the latent variables associated with the role of grader (i.e., 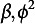 ).

Figure 2 shows an illustrative path diagram for the proposed model under a simplified setting with 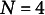 students and 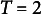 assessments. Compared with many traditional latent variable models, the current path diagram shows a network structure where the latent variables of different individuals interact with each other. This phenomenon is due to the network structure of peer grading data, where each grade involves two students- one as the examinee and the other as the grader.

The proposed model is useful in different ways. First, the model provides a measurement model for the true score of each student *i*’s assessment *t*. By inferring each latent variable, 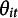 , whose technical details will be discussed in Section 2.3, the grader and assessment effects will be adjusted. Thus, a more accurate aggregated score may be obtained. Second, it allows us to further assess each student’s overall performance and consistency as an examinee by inferring 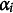 and 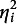 . Third, the model also provides a measurement model for the characteristics of each student as a grader. Specifically, the bias and reliability of each grader can be assessed by inferring 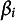 and 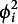 . Such results can be used to reward the best-performing graders and offer feedback to help those who need improvement. Finally, the statistical inference of the structural parameters in 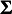 allows us to address substantive questions, such as whether a student who performs better in the coursework tends to be a more reliable grader.

### 2.3 Bayesian inference

We adopt a fully Bayesian procedure for drawing statistical inference under the proposed model.

#### 2.3.1 Prior specification

We first specify the prior for the assessment difficulty parameters 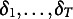 . When *T* is large, we can get reliable estimates of the assessments’ population parameters (e.g., the mean and the variance, Cao & Stokes, 2008; De Boeck, 2008; Fox & Glas, 2001; Gelman, 2006). In such cases, we can use a hierarchical prior specification and assume that 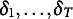 are i.i.d. following a specific prior distribution (e.g., a normal distribution) with some hyper-parameters. Then, we set a hyper-prior distribution for the hyper-parameters. When *T* is small, it is not reasonable to assume to observe a representative sample of assessments, and the estimates at the population level might be very unreliable (De Boeck, 2008). Therefore, we let each 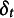 have a weakly informative prior distribution of 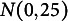 . However, tailored considerations must be made depending on the specific dataset, and different prior specifications might be specified (Gelman et al., 2013).

We specify a prior for the parameters 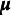 and 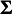 in the joint distribution for the student-specific latent variables. Recall that 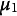 and 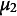 are constrained to zero, so no prior is required. As for 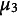 and 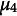 , they are assumed to be independent, and each follows a weakly informative normal prior 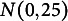 . Finally, for the covariance matrix 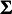, we reparameterize it as 

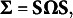

where 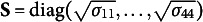 is a 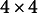 diagonal matrix with diagonal entries the standard deviations of 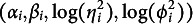 , and 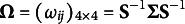 is the correlation matrix of 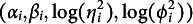 . The prior distribution on 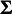 is imposed through the priors on 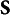 and 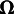. For 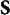 , we assume 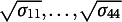 to be i.i.d., each following a half-Cauchy distribution with location 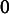 and scale 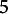 . For the correlation matrix 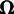, we assume a Lewandowski–Kurowicka–Joe (LKJ) prior distribution with shape parameter 1 (Lewandowski et al., 2009) that corresponds to the uniform distribution over the space of all correlation matrices.

#### 2.3.2 Model comparison

Several reduced models can be derived under the proposed framework as special cases. For instance, a reduced model may be obtained by constraining 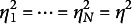 , that is, students’ performance consistency as examinee is constant across individuals. Another reduced model may be derived by constraining 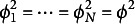 . An even more simplified model can be obtained by imposing both sets of constraints. Given a dataset, Bayesian model comparison methods may be used to find the best-performing model among the full and the reduced models and, thus, provide insights into the peer grading system and yield more accurate aggregated grades.

We consider a Bayesian leave-one-out (LOO) cross-validation procedure for model comparison, which concerns the model’s prediction performance. For a given dataset and a given model, this procedure computes the expected log point-wise predictive density (elpd; Vehtari et al., 2017) to measure the overall accuracy in predicting each data point (i.e., peer grade) based on the rest of the data. More precisely, we define the Bayesian LOO estimate of out-of-sample predictive fit as 

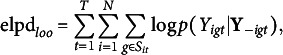

where 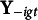 denotes all the observed peer grades except for 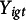 , and 
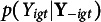
 denotes the conditional probability mass function of 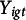 given 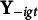 under the fitted Bayesian model. A model with a higher value of 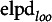 is regarded to have better prediction power and, thus, is preferred. In Section 3, we also report the Watanabe–Akaike information criterion (WAIC), which corrects the expected log point-wise predictive density by adding a penalty term for the effective number of parameters (Vehtari et al., 2017).

#### 2.3.4 Computation

As illustrated in Figure 2, the proposed model involves a latent space with dimension 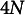 and a complex dependence structure between the observed data and the latent variables. This complex model structure makes its statistical inference computationally a challenge. We use a Markov Chain Monte Carlo (MCMC) algorithm for statistical inference. More specifically, we adopt the No-U-Turn HMC sampler (Hoffman & Gelman, 2014), a computationally efficient MCMC sampler, and implement it under the Stan programming language. Compared with classical MCMC samplers, such as the Gibbs and Metropolis–Hastings samplers, the No-U-Turn HMC sampler uses geometric properties of the target distribution to propose posterior samples. It thus converges faster to high-dimensional target distributions (Hoffman & Gelman, 2014). Further computational details are given in the appendix.

Regarding the implementation, we use the CmdStan interface (Stan Development Team, 2023) for posterior sampling, which is a command-line interface to Stan that is considerably more efficient than using R as the interface. For all the models, four HMC chains are run in parallel for 2,000 iterations, of which the first 1,000 iterations were specified as the burn-in period. We use the rstan R package to analyze the resulting posterior samples, more specifically, it enables us to merge the MCMCs, compute the summary statistics of the posteriors and check the MCMC mixing and convergence. Moreover, the R package loo (Vehtari et al., 2017) and Bayesplot (Gabry et al., 2019) are used separately for model comparisons and to plot the results, respectively. The computation code used in our analysis, the computational time, and other details on model diagnostics are publically available online.^1^

### 2.4 A related model

One of the most well-known approaches to latent variable modeling of peer grading data was proposed by Piech et al. (2013). They present three models of increasing complexity, in which the observed score is assumed to be a function of two independent variables: the student’s ability (also known as the true score) and the effect of the grader (often considered the error part). This type of decomposition is very common in rater effects models (Gwet, 2008; Martinková et al., 2023) and is also assumed in our framework. For comparison purposes, we briefly discuss their more complex model, which is also considered in Section 3 and compared with the one we present in Section 2.2. The notation we adopt in presenting their model is consistent with our framework. They assume that the observed score 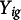 is normally distributed with the mean parameter given by the sum of the true score 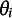 and the grader bias 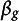 , and the precision parameter being a linear function of the true score of student *g*: 

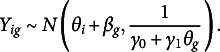

The model assumes that the true scores of students, denoted by 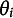 , are independently and identically normally distributed, 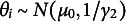 , 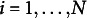 . In addition, the model assumes that graders’ biases denoted by 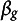 , are i.i.d. normally distributed, 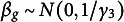 , 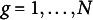 .

While this model relates to the proposed method, the two have several differences. For example, the model proposed by the authors does not account for the difficulty level of the assignment. Even if they propose to use normalized grades (*z*-scores) to remove any assignment effects, it may still be useful to estimate the difficulty level of the assignment. Furthermore, the model assumes that the parameters 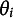 and 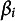 , which are the same student indexes, are independent. It also imposes a strict constraint on the precision parameter of the observed score. Specifically, it does not allow the precision to vary given the same value of 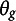 , and it assumes that the precision is independent of the grader bias 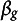 . Finally, the model does not account for the temporal dependency in the presence of multiple assessments. This model, denoted in Section 3 as PM (i.e., Piech’s Model), is compared against the proposed one using real data from multiple and single assessment contexts.

### 2.5 Reduced model for a single assessment

Some peer grading data only involve a single assessment, as the case for one of our real data examples in Section 3. The proposed model can still be applied in that situation, but certain constraints must be imposed for model identification. Details of the Bayesian inference for this model are given in the appendix.

#### Modeling peer grade 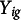


2.5.1

With only one assessment, the notation for peer grade simplifies to 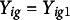 , and its decomposition simplifies to 
(3)

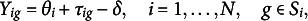

where the subscript *t* is removed from all the notations in (1), and the interpretation of the variables remains the same. Due to the lack of multiple assessments, the examinee parameters 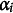 and 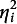 in the main model, Equation (2), can no longer be identified and, thus, are not introduced here.

#### Modeling grader effect 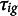


2.5.2

Each student *g* grades the assessment of multiple peers. Let 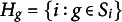 be the peers whose work is graded by student *g*. It is assumed that 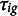 , 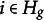 , are i.i.d., following a normal distribution 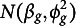 . The interpretation of these parameters is the same as in the primary model (see Section 2.2).

#### 2.5.3 Joint modeling of student specific latent variables

**Figure 3:**
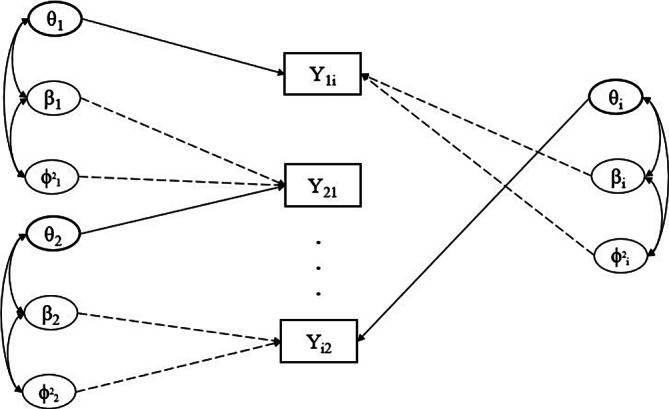
Path diagram representing the network structure of peer grading data for a single assessment. *Note*: The latent variables of three independent students are represented as an example. The box indicates the students’ grades for a single assessment. The double arrows represent correlation, while the straight (solid and dotted) lines represent the effect of the respective latent variable. The meaning of the arrows is consistent with those of Figure 2. The solid line represents the effect of the latent variable related to the role of the examinee (i.e., 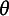 ). The dotted lines refer to the effect of the latent variables associated with the grader role (i.e., 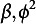 ).

The reduced model involves three student-specific latent variables 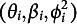 . Similar to the main model, we assume that 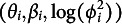 , 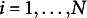 , are i.i.d., each following a multivariate normal distribution 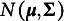, where 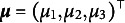 and 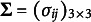. Similar to the main model, we constrain 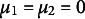 , while keep 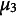 freely estimated. Figure 3 gives an illustrative path diagram for this reduced model with 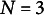 students.

## 3 Real data examples

Two real-world applications referring to single- and multiple-assessments settings are considered here. Various models are compared for each dataset, and the one that exhibits the best predictive performance is used for inference.

### 3.1 Multiple-assessments setting

The peer grading data are from Zong et al. (2021). In this data, 274 American undergraduate students taking a Biology course completed four double-blind peer gradings throughout the course ( 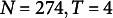 ). The assessments had a similar format, and the online peer reviewing system managed the submission and peer grading procedures *SWoRD/Peerceptiv* (Patchan et al., 2016). Students’ mean age was 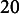 , and 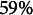 were female. Students’ ethnicity was quite heterogeneous, 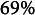 were Asian, 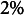 Black, 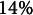 Latinx, and 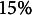 White. On average, each work was graded by a random set of five other students. Zong et al. (2021) produced the peer grading score as the average across different rubrics. As a result, gradings are on a 1–7 continuous scale. The minimum and the maximum observed values were, respectively, 1 and 7. The mean and the median grades were 5.414 and 5.500, respectively, which suggest that data are slightly negatively skewed. To implement the main model, only students who completed at least three assessments were included in the analysis, which resulted in a sample size of 212 students.

#### 3.1.1 Model comparison

Four different models of increased complexity are fitted and compared. In the first model (M1), we only accounted for one student-specific latent variable: the ability and the assessment difficulty level. This model did not consider the effects of graders, such as their systematic biases and reliability levels. Additionally, M1 assumed that the student’s ability was equal across all assessments. This is the more constrained model. In the second model (M2), we relax our assumptions and consider the graders’ effects, such as their systematic bias and reliability levels. To do this, we use a three-dimensional multivariate normal distribution to jointly model the student-specific latent variables, including 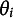 , 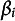 and 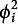 , 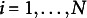 . It is worth noting that fitting M2 is like fitting the reduced model for a single assessment separately (see Section 2.5), except that students are assumed to have the same ability level across assessments, that is, 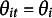 , 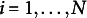 . In the third model (M3), we relax this assumption and allow for variations in students’ abilities across assessments by introducing a fourth student-specific latent variable, 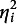 , 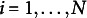 . Under this model, examinee- and grader-specific latent variables, respectively, 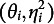 and 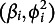 are assumed to be independent. This assumption is relaxed in the fourth model (M4) in which the latent variables 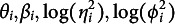 , 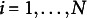 , are allowed to be correlated. M4 is the main model introduced in Section 2.2. We also compare these models with the one proposed by Piech et al. (2013) and detailed in Section 2.4. Under this multiple-assessments setting, we let the difficulty parameter vary across assessments for comparison purposes.

Model comparison is based on the predictive performance criteria discussed in Section 2.3. The models are fitted using the prior specifications and posterior procedure discussed in Section 2.3. Grades are on a continuous 1–7 scale, with the midpoint considered the average assessment difficulty. Therefore, we have set the prior distribution for 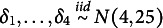 . The students are then given an estimate of the true score for each work and a reliability estimate as a grader.

#### 3.1.2 Results from the selected model

Upon graphical inspection of the MCMCs, no mixing or convergence issues were detected, as indicated by 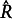 values being less than 1.01. The Number of Effective Sample Size was above the cut-off 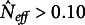 for all the structural parameters (Gelman et al., 2013). The average computational time per chain varies from 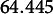 to 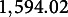 s (seconds), respectively, recorded for models M1 and M4. Further details on model diagnostics (e.g., trace plot, 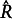 , 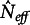 , convergence diagnostic plots), as well as computational time, can be found in Supplementary Material.^2^

Table 1 gives the value of the LOO expected log point-wise density elpd 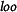 and the relative standard error for each model fitted, including the pairwise difference in terms of elpd 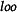 between M4 and each of the other models; in the last column we also report the WAIC (Gelman et al., 2013). The procedure for model comparison showed that M4 provides the best predictive performance. The slightly better performance of M4 over M3 in terms of these criteria supports our assumption of the examinee- and grader-specific latent variables being correlated.

**Table 1:** Multiple-assessments example: Four model specifications are compared using a leave-one-out cross-validation approach

Model	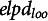	*SE*	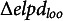	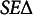	WAIC
M4	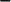 3,751.8	49.9	–	–	7,358.18
M3	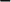 3,770.0	49.3	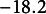	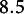	7,382.30
PM	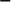 3,937.7	52.0	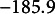	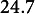	7,819.19
M2	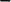 3,939.4	54.1	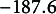	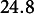	7,820.01
M1	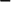 4,470.7	54.2	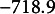	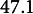	8,939.57

*Note*: The expected log point-wise density value (elpd 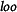 ) and its respective standard error ( 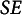 ) are reported. The models are given in descending order based on their elpd 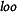 values. 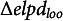 gives the pairwise comparisons between each model and the model with the largest elpd 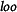 (M4), and 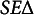 is the standard error of the difference. The Watanabe–Akaike information criterion (WAIC) is given in the last column for each model.

Table 2 shows that the difficulty levels of the assessments are increasing throughout the course. The 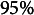 quantile-based credible intervals of the assessment difficulty parameters are moderately narrow, indicating a low level of uncertainty for these parameters.

**Table 2:** Multiple-assessments example: Model M4 estimated structural parameters

	Parameter	Post. Mean	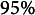 CI
Assessments	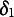	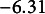	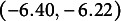
	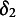	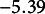	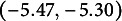
	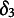	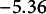	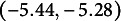
	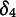	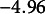	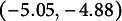
Students	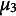	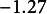	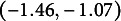
	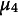	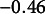	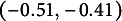
	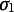	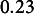	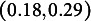
	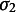	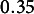	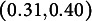
	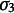	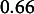	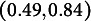
	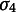	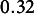	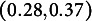
	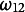	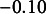	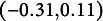
	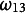	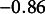	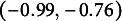
	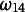	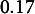	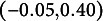
	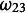	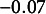	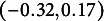
	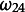	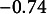	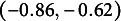
	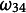	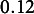	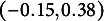

*Note*: The posterior mean (Post. Mean) and the 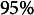 quantile-based credible interval (CI) are reported for each parameter. The parameter 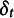 represents the difficulty level of the assessment *t*; 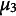 and 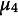 are the location parameters of the third and the fourth latent variable, respectively; 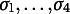 are the standard deviations of the latent variables; 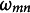 is the correlation parameter between the latent variables *m* and *n*.

The posterior means for the latent variable variances are 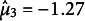 with a 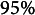 credible interval of 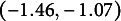 and 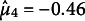 with a 95% credible interval of 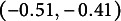 . This implies that, on average, the variance of the student’s ability is smaller than the error variance of the grades they give. In other words, they are more consistent as an examinee than a grader. This seems reasonable, considering that they are not grader experts. Note that these parameters are expressed on a logarithmic scale, meaning that the average variance of the students’ proficiency across different assessments is 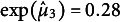 , and, on average, their reliability parameter is 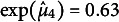 .

Students are moderately homogeneous regarding their mean abilities, as suggested by 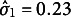 . In contrast, they are more variable in their systematic bias, 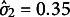 . In other words, they are, on average, more similar as examinees than as graders. Moreover, students are widely different concerning their consistency across assessments, 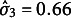 . Finally, they have slightly less variability concerning the reliability parameters as indicated by 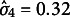 .

Regarding the dependencies among the latent variables, higher values of students’ proficiency are associated with higher consistency values. Indeed, there is evidence of a strong correlation between the first and the second student-specific latent variable, respectively, 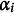 and 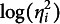 , as suggested by 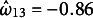 and the 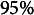 credible interval of and 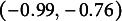 . In addition, higher mean bias values are associated with higher reliability levels. This is evidenced by 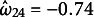 and the 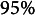 credible interval of 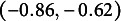 . The estimates of the other correlation parameters do not provide clear evidence about any other dependencies. The grader’s effect explains, on average, 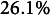 of the grading variance, conditioning on the assessment difficulties.

**Figure 4:**
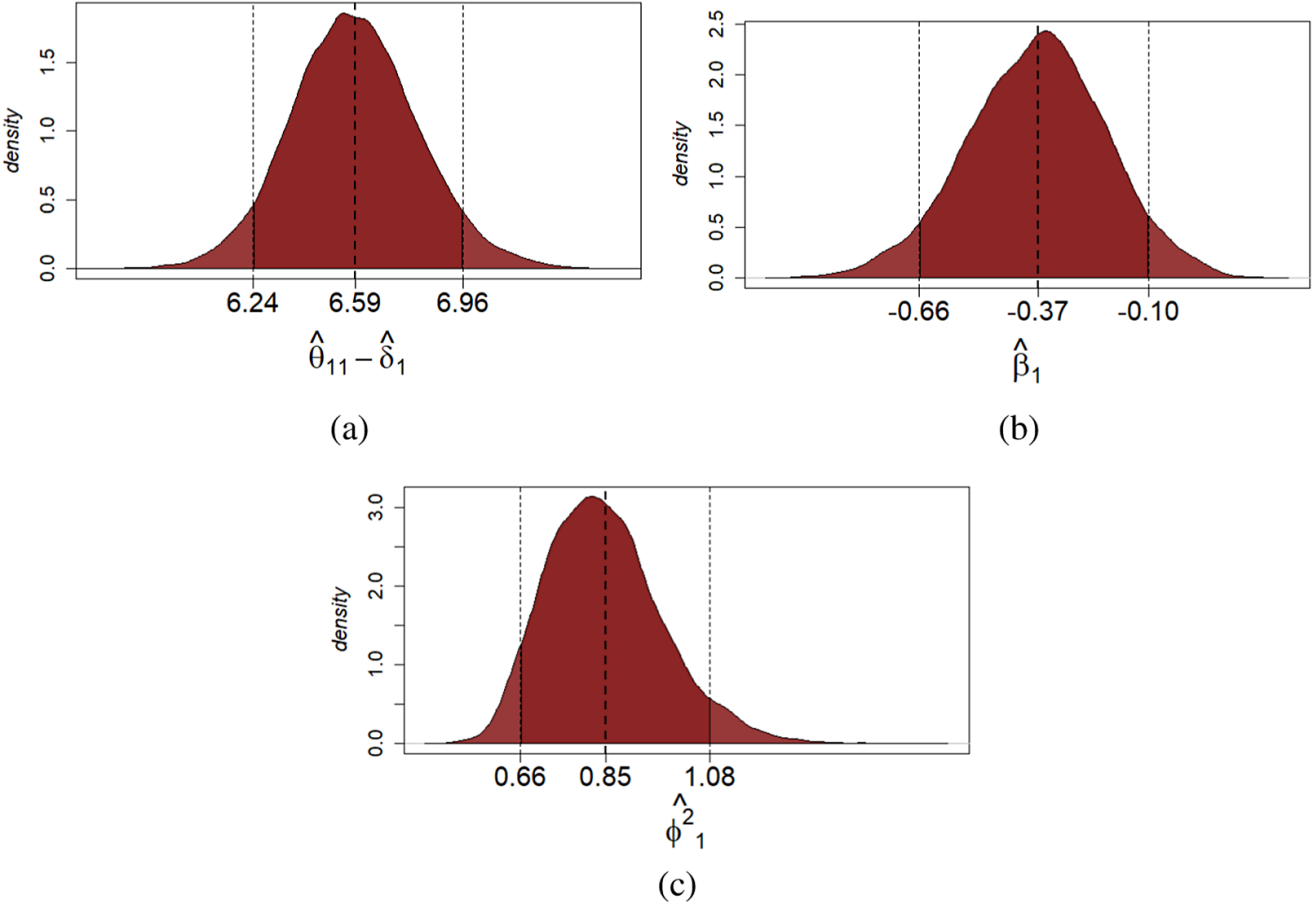
Multiple-assessments example: Posterior distribution of the true score of the first assessment (a), mean bias (b), and reliability (c) of student 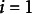 . *Note*: The black dotted lines indicate the 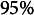 quantile-based credible interval and the posterior mean of each estimated parameter.

At the student-specific level, a score estimate and a 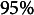 quantile-based credible interval may be provided for each assessment to measure the uncertainty. For students’ scores, the posterior mean of 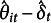 can be used as a point estimate. Additionally, the posterior distributions for both the average bias and the reliability of each grader can be useful in assessing their grading behavior. If a grader is accurate and reliable, their 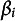 and 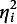 values should be close to zero. Conversely, values far from zero indicate biased and unreliable grading behavior. Both parameters are provided with a 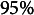 quantile-based credible interval. For illustrative purposes, the posterior estimates of the true score 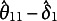 , the mean bias 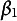 and the reliability 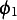 of student 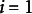 are reported in Figure 4. On the examinee side, the posterior estimates of the true score suggest that for the first assessment 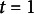 , the proficiency level of this student is slightly larger than the average. On the grader side, based on the posterior estimates of 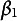 and 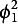 , this student is more severe and moderately less reliable than the average (note that 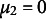 and the posterior mean of 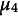 is 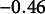 on the log scale). Additional results about the posterior mean estimates of the student-specific latent variables, including their density plots, pairwise scatter plots, and Pearson correlations between latent variables, are presented in Supplementary Material. According to these results, the posterior mean estimates seem well-behaved, based on which the multivariate normality assumption of the latent variables does not seem to be severely violated.

### 3.2 Single assessment setting

The data used for the cross-sectional analysis were obtained from an applied economics undergraduate course at the University of Oviedo, as reported by Luaces et al., (2018). The sample consisted of 108 students who participated in a double-blind individual peer assessment on an online platform provided by the university. Each coursework was an open-response assessment rated by ten students according to different rubrics on Likert scales of various lengths. For the present analysis, we consider the sum of the ratings given on these other aspects as the final grade. The observed grades ranged from 0 to 12, with a mean of 7.526 and a median of 8.000. Further information on the grading procedure might be found in Luaces et al. (2018).

#### 3.2.1 Model comparison

Four models are fitted and compared. Three are nested models, and the fourth is the model provided by Piech et al. (2013) and discussed in Section 2.4. In the first model (M1), we specify one single student-specific latent variable: the student ability and the assessment difficulty parameter. This model did not consider the effects of graders, such as their systematic biases and reliability levels. In other words, graders’ mean bias is fixed to zero, and they are assumed to be equally reliable. This model is the same as the (M1) model detailed in Section 3.1, but only with one assessment. In the second model (M2), we let the graders’ mean biases be freely estimated. Moreover, we let this second student-specific latent variable correlate with the first one. Indeed, they are assumed to be i.i.d. across students, following a bivariate normal distribution. In the third model (M3), we relax the assumption of equal reliability across different graders. However, the latent ability 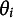 is assumed to be independent of the other features of the student as a grader (i.e., 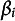 and 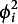 ), for 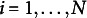 . This independence assumption is relaxed in the fourth model (M4) and we allow them to be correlated. M4 is the model presented in Section 2.5. The models are fitted using the prior specifications and the posterior procedure discussed in Section 2.3. As with the multiple-assessments example, the prior distribution for the difficulty parameters is set to 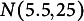 . The students are then given an estimate of the true score for each assessment and a reliability estimate as a grader.

#### 3.2.2 Results for the selected model

As with the multiple-assessments example, no mixing or convergence issues were detected, as indicated by 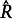 values less than 1.01. The average computational time per chain ranges from 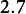 to 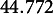 s, respectively, recorded from Models M1 and M4. Further details on Model diagnostics (e.g., trace plot, 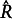 ), as well as computational time, can be found in the Appendix and an online repository.^3^

Table 3 indicates that model M4 has the best predictive performance, though its advantage over M3 is very small. 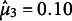 gives the mean graders’ reliability level (i.e., the posterior mean of 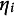 ), and there is considerable variability among them as indicated by 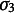 . Indeed, the estimates of 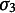 on a log scale imply a posterior standard deviation of 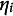 larger than one on the original rating scale.

**Table 3:** Single assessment example: Four model specifications are compared using a leave-one-out cross-validation approach

Model	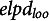	*SE*	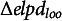	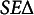	WAIC
M4	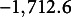	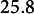	0.0	0.0	3,410.29
M3	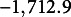	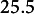	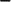 0.3	0.7	3,410.76
M2	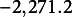	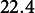	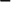 558.7	19.9	4,535.97
M1	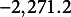	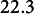	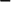 558.7	19.8	4,536.05
PM	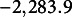	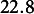	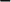 571.4	20.8	4,557.39

*Note*: The expected log point-wise density value (elpd 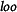 ) and its respective standard error ( 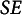 ) are reported. The models are given in descending order based on their elpd 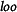 values. 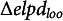 gives the pairwise comparisons between each model and the model with the largest elpd 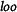 (M4), and 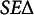 is the standard error of the difference. The Watanabe–Akaike information criterion (WAIC) is given in the last column for each model.

**Table 4:** Single assessment example: Model M4 estimated structural parameters

	Parameter	Post. Mean	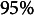 CI
Assessment	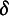	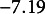	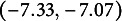
Students	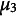	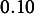	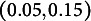
	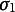	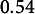	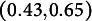
	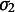	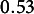	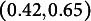
	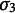	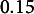	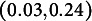
	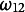	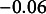	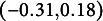
	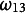	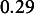	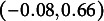
	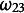	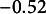	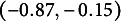

*Note*: The posterior mean (Post. Mean) and the 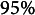 quantile-based credible interval (CI) are reported for each parameter. The parameter 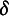 represents the difficulty level of the assessment; 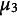 is the location parameter of the third latent variable; 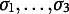 are the standard deviations of the latent variables; 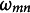 is the correlation parameter between the latent variables *m* and *n*.

Students are very similar in their latent ability, as suggested by the small values of the posterior standard deviation of their abilities, that is, 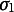 (see Table 4). The same extent of variability is estimated concerning their mean biases 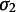 . This implies that students are pretty homogeneous regarding proficiency in doing the assessment and severity in grading their peers. The 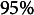 CI for the correlation parameters 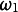 and 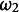 do not suggest a clear relation between the respective latent variables. A positive correlation between graders’ bias and their reliability is highlighted by the estimate of 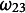 . Nonetheless, the relative credible interval is quite large, implying uncertainty about the correlation size. Grader’s effects explain, on average, the 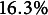 of the grading variance.

**Figure 5:**
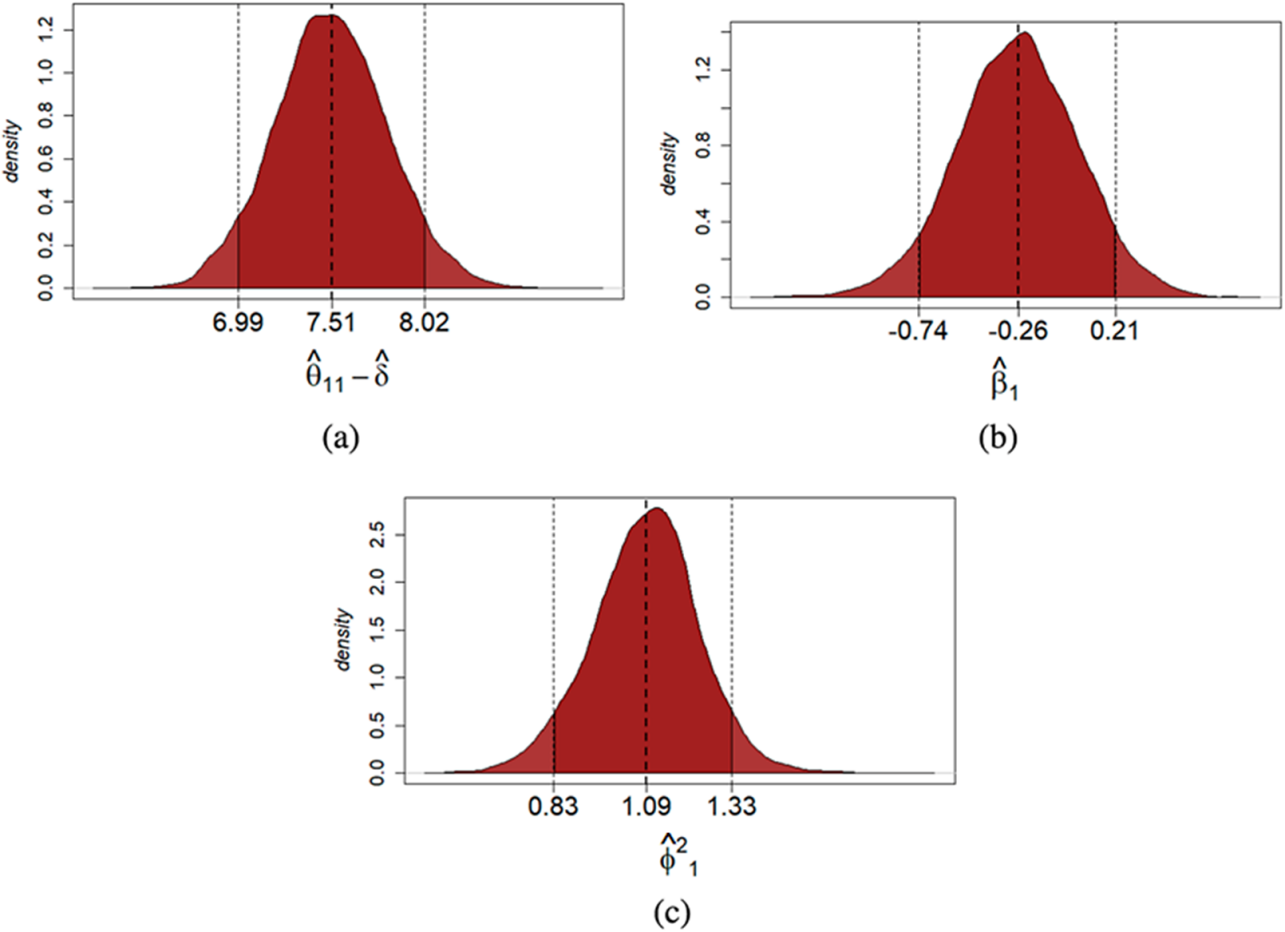
Single assessment example: Posterior distribution of the true score (a), mean bias (b), reliability (c) of student 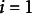 . *Note*: The black dotted lines indicate the 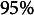 quantile-based credible interval and the posterior mean of each estimated parameter.

Each student might receive a true score estimate at the individual level. The posterior mean of 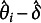 might be used as a point estimate for students’ true scores. Moreover, the posterior distributions of both the mean bias and the reliability of each grader might be helpful information to assess their grading behavior. As an illustration, the posterior estimates of the true score 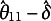 , the mean bias 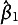 and the reliability 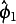 of student 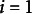 are reported in Figure 5. On the examinee side, the true score’s posterior estimates suggest that this student’s proficiency level is slightly larger than the average. On the grader side, the posterior estimates of 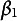 and 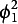 suggest that this student is moderately more severe than the average in terms of mean bias but average in terms of reliability level (note that 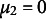 and the posterior mean of 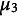 is 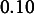 on the log scale).

## 4 Discussions

This article presents a new modeling framework for peer grading data, which introduces latent variables to capture the dependencies in the data from the network structure of peer grades and the dual role of each student as an examinee and a grader. The statistical inference uses a Bayesian method, and an algorithm based on the No-U-Turn HMC sampler was developed. The proposed model was applied to two real-world peer grading datasets, one with a single assessment and the other with four. The results showed that the proposed model had superior prediction performance in real-world applications and that the MCMC did not suffer from mixing or convergence issues.

The current work also has some limitations. First, the peer grades in the applications in Section 3 are bounded, which may cause ceiling and floor effects, as the variability of student performance is no longer measurable when they receive a very high or low score. However, the proposed model is based on several normal assumptions, which fail to capture such phenomena. To model bounded grades, we may add a nonlinear transformation to the right-hand side of (1) to ensure 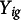 to be bounded.

Second, it is not easy to verify the assumptions about the latent variables in our model and further validate their interpretation as we cannot observe the latent variables. Specifically, the multivariate normality assumption about the student-specific latent variables may be quite strong for many real-world settings. As pointed out by Ma & Genton (2010), severe violation of this assumption can lead to substantially biased estimates. Nevertheless, our sensitivity analysis in the Supplementary Material shows that the model estimates are still reasonably accurate under mild deviations from the normality assumption. Without additional information, it is hard to disentangle different assumptions about the latent variables and verify them separately. We can only check whether the model-implied distribution for the observed data fits its empirical distribution (e.g., using Bayesian LOO and WAIC) and use it to compare different models. Using this approach, we can only tell that the assumptions of one model are more sensible than those of the other. To further verify our model’s assumptions, we may collect data with both peer and instructor grades. The instructor’s grades may be used as the underlying truth to check some specific assumptions in our model.

The current work can be further extended in several directions. First, in formative assessment settings, people are often interested in the growth of students over multiple assessments during a course. Therefore, extending the proposed model to a longitudinal setting and developing a latent growth curve model for peer grading may be helpful. To explore this direction, we have considered a simple extension of the proposed model and performed a small simulation study in Appendix B. This model assumes the true score 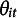 to follow a latent growth curve model. While this model performed well in the simulation, it may be oversimplified for real-world settings. In practice, student characteristics as a rater and the difficulty levels of the assessments may also change over time. Simultaneously modeling all these changes may result in model identification issues. We leave this problem for future investigation.

Second, additional context information, such as student- and classroom-related factors, is often available in formative assessment settings. Such information is useful in explaining and predicting each student’s performance both as an examinee and a rater. In this regard, we believe extending the framework of explanatory item response models (Kim & Wilson, 2020; Wilson & De Boeck, 2004) to the current setting to include context information as covariates is useful.

Third, the reliability of the peer grading system based on the proposed model is worth investigating. This may be done by adapting the generalizability theory (Brennan, 2001, 2010), originally established under the traditional cross-classified random effects models, to the current model. With the new generalizability theory, we may evaluate the reliability of the system from different perspectives (e.g., examinees, raters, and assessments). Moreover, while the average reliability level of graders might affect the accuracy of the score estimates, a larger number of graders per student’s assignment might mitigate this effect and improve their accuracy (see the Supplementary Material for additional simulation results about these aspects).

Fourth, many real-world peer grading systems involve ordinal peer grades. The proposed model may be extended to ordinal data by replacing the linear model (1) with a generalized linear model. One possible formulation is given in Appendix D, which still mimics the proposed model but replaces (1) with a partial credit model form (Masters, 1982). Alternative models also may be available, such as one based on the graded response model (Samejima, 1969). The suitability of these models for peer grading remains to be studied through a theoretical investigation and numerical studies based on simulated and real data. We leave it for future investigation.

Finally, it should be noted that although the Bayesian approach allows for statistical inferences, it can be time-consuming to compute. The high computational cost is due to the proposed model’s high dimensionality, which depends on the number of students and assessments. To make the proposed method scalable for large-scale applications, like MOOC data, advanced computational methods for Bayesian inference, such as stochastic gradient MCMC algorithms, should be explored (Nemeth & Fearnhead, 2021).

## Supporting information

Mignemi et al. supplementary material Mignemi et al. supplementary material

## Figures and Tables

**Figure B1:**
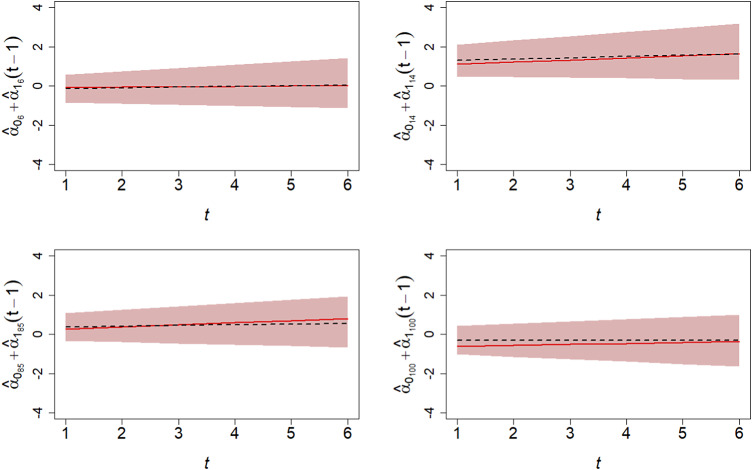
Estimated linear latent growth of a random sample of four students. *Note*: The red and the black dotted lines are the true linear growth and the estimated one, respectively; the red bands are the 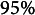 credible intervals of this trend.

**Figure C2:**
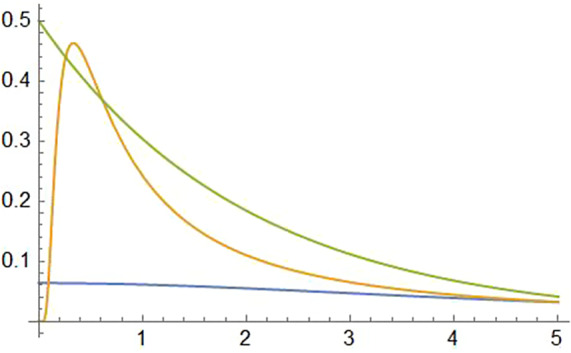
Priors placed under different scenarios on 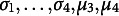 . *Note*: The blue, orange, and green solid lines indicate, respectively, the half-Cauchy, the inverse-gamma and the exponential priors.

## Footnotes

1 Our code is available through the link: https://osf.io/v3ucw/?view_only=aad3bc91cbda43cc9e6c490409323839.

2 Available through the link https://osf.io/v3ucw/?view_only=aad3bc91cbda43cc9e6c490409323839.

3 Available through the link: https://osf.io/v3ucw/?view_only=aad3bc91cbda43cc9e6c490409323839.

## Supplementary material

The supplementary material for this article can be found at https://doi.org/10.1017/psy.2025.10021.

## A Computational details

To resolve the convergence issue and make the MCMC mix well, we used a non-centred reparametrization for the multivariate normal distribution (Betancourt & Girolami, 2015; Gelman et al., 2013; Papaspiliopoulos et al., 2007). We express the distribution of the vector of student-specific latent variables through an affine transformation, such that

Here,  is the Cholesky factor of the correlation matrix  and  is the diagonal matrix of the standard deviation of the latent variables, ,  ; note that . The element of the four-dimensional vector  are i.i.d. following a standard normal distribution,  ,  . Notice that, as stated above,  for identifiability purposes.

Under this inference procedure, each parameter and student-specific latent variables might be provided with a posterior point estimate, for example, the posterior mean, and an interval estimate, for example, a  quantile-based credible interval. The latter might be seen as an uncertainty measure of the estimated parameter; broader intervals suggest more uncertainty about the values of the parameters, whereas narrower intervals reflect less uncertainty about their values.

## B Extension to latent growth curve model

### B.1 Model specification

When sufficient assessments are given over time, evaluating students’ growth during that period may be interesting. This can be done using Latent Growth Curve (LGC) modeling (Bollen & Curran, 2006). For example, a linear latent curve unconditional model can expand the structural model (2) for true score  by assuming

(B.1)

where  ,  , and  are student-specific latent variables, and  ,  are a pre-specified coding of time. The linear function  can be interpreted as the latent trajectory of student *i*. The coding of time can be chosen based on the time when the assessments are given. In the special case when the assessments are given at equally spaced intervals, we can set  . We keep the model for  unchanged. The student-specific latent variables now include  . In line with our assumptions in the main model, we assume that  ,  are i.i.d., and follow a multivariate normal distribution.

This model can be further extended to capture nonlinear trajectories. For example, a quadratic latent curve model may be assumed for  by assuming

(B.2)

where  ,  ,  , and  are student-specific latent variables, and  ,  are still a pre-specified coding of time. The random second-order coefficient  is the rate of change in the linear component along time and represents the acceleration in growth for student *i*’ ability (Biesanz et al., 2004; Bollen & Curran, 2006). The joint model for student-specific latent variables can be extended accordingly.

The prior specifications discussed in Section 2 for the unknown parameters,  and , might be consistently adopted for the current model. The same procedures and reparametrization used for the posterior computation introduced above might be freely applied here for the multivariate normal distribution. The Bayesian model comparison procedure discussed in Section 2 might be used to compare the models under the main and LGC frameworks. As for the previous models, each parameter and student-specific latent variables might be provided with a posterior point estimate, for example, the posterior mean, and an interval estimate, for example, a  quantile-based credible interval. Indeed, it might be seen as an uncertainty measure of the relative estimated parameter.

### B.2 A simulation under the LGC model

Homoscedastic LGC models typically require at least three time-points per individual (e.g., Curran et al., 2010), whereas, our proposal concerns a heteroscedastic setting that allows some variance terms to be individual-specific and implies a larger number of parameters. Given the relatively small number of assignments per student (at most four) and the rather small sample size  , the LGC model might not be suitable for the real data analyzed in Section 3.1. We provide a simulated example based on the above LGC model an illustrative example.

#### B.2.1 Data generation

We generated a dataset from the linear LGC model in which a sample of  students are assigned  assessments. For each assessment, each student’s work is graded by a random subset of other  students. The following values are fixed for the structural parameters of the model:  ,  , ,  is a five-dimensional identity matrix, , see Table C2.

**Table C2:** Estimated structural parameters

	Parameter	True value	Post. Mean	CI
Assessments				
				
				
				
				
				
				
				
Students				
				
				
				
				
				
				
				
				
				
				
				
				
				
				
				
				
				

*Note*: The true value, the posterior mean (Post. Mean) and the  quantile-based credible interval (CI) are reported for each parameter. The parameter  is the difficulty level of the assessment;  ,  , and  are the location parameters of the second, the fourth and the fifth latent variable, respectively;  are the standard deviations of the latent variables;  is the correlation parameter between the latent variables *m* and *n*.

#### B.2.2 Estimated parameters

All the considerations on the prior and computational aspects discussed in the main text are consistently followed here. The graphical inspection of the MCMCs does not suggest any mixing or convergence issues, which is consistent with the low values of  . The average computational time per chain recorded is  s.^4^

The estimates of the structural parameters are reported in Table C2. All the true values are included in the  credible intervals, even though these posterior intervals are considerably large. This uncertainty might be due to the small sample size  . The only exception is the correlation parameter  , whose  credible interval does not include the true value (even if it is the difference between the upper bound of the interval and the true value is practically negligible). More replications might shed light on these aspects.

Under this model, along with the estimates of the true grade and the grader’s effects (i.e., the mean bias and the reliability), each student might be provided with the estimate of his/her latent linear growth trend. For illustrative purposes, we plot the estimated growth of four students in Figure B1. Note that the  credible intervals of the student-specific trend  are proportional to the values of *t*. This is because the  and the  quantiles of the posterior of  are multiplied by this covariate.

## C Prior sensitivity analysis

We performed a prior sensitivity analysis to investigate the impact of the prior on final model estimates (Gelman et al., 2013). As discussed by Gelman (2006), inferences might be very sensitive to the choice of the prior distribution for hierarchical variance parameters, and valuable information might come from tailored stimulative studies. As a preliminary analysis, we fit the main model on different generated datasets comparable to the real dataset analyzed in Section 3.1. For each dataset, we alternatively fit the main model under three prior specifications, resulting in three different scenarios. We compare the respective estimates through the root mean square error (RMSE) and the mean absolute error (MAE).

### C.1 Data generation

We generated  independent datasets from the main model in which a sample of  students are assigned  assessments. Each student’s work is graded by a random subset of other  students for each assessment. The following values are fixed for the structural parameters of the model:  ,  , ,  is a four-dimensional identity matrix, .

**Table C3:** Root mean square error (RMSE) and mean absolute error (MAE) related to students’ true scores and structural parameters under different scenarios across 10 independent datasets

Parameter	Scenarios					
	*1*		*2*		*3*	
	RMSE	MAE	RMSE	MAE	RMSE	MAE
	0.243	0.195	0.244	0.196	0.244	0.196
	0.182	0.138	0.182	0.137	0.183	0.138
	0.159	0.138	0.161	0.142	0.159	0.138
	0.131	0.095	0.131	0.096	0.131	0.095
	0.045	0.037	0.048	0.037	0.046	0.038
	0.036	0.029	0.037	0.029	0.036	0.029
	0.068	0.061	0.070	0.062	0.069	0.061
	0.100	0.087	0.097	0.082	0.099	0.085
	0.081	0.069	0.117	0.109	0.083	0.069
	0.065	0.045	0.080	0.058	0.067	0.046
	0.135	0.108	0.133	0.106	0.131	0.106
	0.038	0.029	0.025	0.019	0.036	0.028
	0.043	0.037	0.039	0.033	0.042	0.037
	0.029	0.016	0.022	0.011	0.028	0.015
	0.044	0.033	0.041	0.030	0.044	0.033
	0.005	0.003	0.003	0.002	0.004	0.003
True score	0.591	0.349	0.591	0.349	0.591	0.349

### C.2 Scenarios and priors

We place three different priors on the scale parameters  and on the means  . Under the first scenario, they are assigned a half-Cauchy as recommended by Gelman et al. (2013),  and  . This class of priors are referred to as “weekly informative” by Gelman (2006) because of the gentle slope of their tails, which can let the data dominate the posterior if the likelihood is strong in that region. In the second scenario, we place an inverse-gamma on those parameters,  and  . Under the third scenario, they are assigned an exponential prior,  and  . As shown by Figure C2, the probability density is more spread and diffused under the first scenario as a result of the half-Cauchy prior specification.

### C.3 Results

Inferences are the same across different scenarios, suggesting a robustness of the model to different prior specifications for the scale parameters. Table C3 gives the RMSE and the MAE for the structural parameters and the true scores (i.e.,  ). The RMSE and the MAE of the estimates of the true scores are, on average, 0.59 and 0.34 under all the scenarios. The RMSE and the MAE of the aggregated score using the mean to derive the final grade for each student’s work (Reily et al., 2009; Sajjadi et al., 2016) are, respectively, 2.46 and 1.98. This suggests that our proposal might consistently mitigate graders’ systematic bias and unreliability.

## D An extension to ordinal peer grades

The models in this article are for continuous grades, while grades in many peer grading systems are on an ordinal scale. In what follows, we discuss how the proposed model may be extended to ordinal grades. We consider a formulation based on the partial credit model (Masters, 1982) under the same setting as our main model in Section 2.2 except for the ordinal grades. More specifically, suppose that  . For each  , we assume that

(D.1)

where  contains the item-specific parameters and the rest of the variables can be interpreted similarly as in Section 2.2. More specifically,  may be interpreted as student *i*’s true score for assessment *t*. The larger the value of  , the more likely it is that  takes a value in a higher category. The variable  can still be interpreted as rater *g*’s bias, as raters with a larger  value tend to give a higher grade on average. In addition,  still indicates rater *g*’s reliability. When  goes to infinity and the rest of the parameters remain fixed, the probability in (D.1) will converge to 0.5, and thus, the probability of  will converge to  , for each category *k*, regardless what the true score  is. In other words, the grade  becomes a purely random guess. On the other hand, when  goes to zero, the distribution of  will concentrate on one of the categories.

Similar to the model in Section 2.2, we can assume  to follow (2) and further set priors for the student-specific latent variables, as well as the rest of the model parameters. Bayesian inference can then be performed.

## References

[r1] Alqassab, M. , Strijbos, J.-W. , Panadero, E. , Ruiz, J. F. , Warrens, M. , & To, J. (2023). A systematic review of peer assessment design elements. Educational Psychology Review, 35, 18.

[r2] Betancourt, M. , & Girolami, M. (2015). Hamiltonian Monte Carlo for hierarchical models. In S. K. Upadhyay , U. Singh , D. K. Dey , & A. Loganathan (Eds.), Current trends in Bayesian methodology with applications (pp. 79–101). CRC Press.

[r3] Biesanz, J. , Deeb-Sossa, N. , Papadakis, A. , Bollen, K. , & Curran, P. (2004). The role of coding time in estimating and interpreting growth curve models. Psychological Methods, 9, 30–52. 15053718 10.1037/1082-989X.9.1.30

[r4] Bollen, K. A. , & Curran, P. J. (2006). Latent curve models: A structural equation perspective. John Wiley & Sons.

[r5] Brennan, R. L. (2001). Generalizability theory. Springer.

[r6] Brennan, R. L. (2010). Generalizability theory and classical test theory. Applied Measurement in Education, 24(1), 1–21.

[r7] Cao, J. , & Stokes, L. (2008). Bayesian IRT guessing models for partial guessing behaviors. Psychometrika, 73, 209–230.

[r8] Casabianca, J. M. , Junker, B. W. , & Patz, R. J. (2016). Hierarchical rater models. In W. J. van der Linden (Ed.), Handbook of item response theory (pp. 477–494). Chapman and Hall/CRC.

[r9] Curran, P. , Obeidat, K. , & Losardo, D. (2010). Twelve frequently asked questions about growth curve modeling. Journal of Cognition and Development: Official Journal of the Cognitive Development Society, 11, 121–136. 21743795 10.1080/15248371003699969 PMC3131138

[r10] De Boeck, P. (2008). Random item IRT models. Psychometrika, 73, 533–559.

[r11] DeCarlo, L. T. , Kim, Y. , & Johnson, M. S. (2011). A hierarchical rater model for constructed responses, with a signal detection rater model. Journal of Educational Measurement, 48(3), 333–356.

[r12] Double, K. S. , Mcgrane, J. A. , & Hopfenbeck, T. N. (2020). The impact of peer assessment on academic performance: A meta-analysis of control group studies. Educational Psychology Review, 32, 481–509.

[r13] Fox, J.-P. , & Glas, C. A. (2001). Bayesian estimation of a multilevel IRT model using Gibbs sampling. Psychometrika, 66, 271–288.

[r14] Gabry, J. , Simpson, D. , Vehtari, A. , Betancourt, M. , & Gelman, A. (2019). Visualization in Bayesian workflow. Journal of the Royal Statistical Society Series A, 182, 389–402.

[r15] Gamage, D. , Staubitz, T. , & Whiting, M. (2021). Peer assessment in MOOCs: systematic literature review. Distance Education, 40(2), 1–22.

[r16] Gelman, A. (2006). Prior distributions for variance parameters in hierarchical models (comment on article by Browne and Draper). Bayesian Analysis, 1(3), 515–534.

[r17] Gelman, A. , Carlin, J. , Stern, H. , Dunson, D. B. , Vehtari, A. , & Rubin, D. B. (2013). Bayesian data analysis. Chapman and Hall/CRC.

[r18] Gin, B. , Sim, N. , Skrondal, A. , & Rabe-Hesketh, S. (2020). A dyadic IRT model. Psychometrika, 85(3), 815–836. 32856271 10.1007/s11336-020-09718-1

[r19] Goldstein, H. (1994). Multilevel cross-classified models. Sociological Methods & Research, 22(3), 364–375.

[r20] Gwet, K. L. (2008). Computing inter-rater reliability and its variance in the presence of high agreement. British Journal of Mathematical and Statistical Psychology, 61(1), 29–48. 18482474 10.1348/000711006X126600

[r21] Han, C. (2018). Latent trait modelling of rater accuracy in formative peer assessment of English-Chinese consecutive interpreting. Assessment and Evaluation in Higher Education, 43(6), 979–994.

[r22] Hoffman, M. D. , & Gelman, A. (2014). The No-U-Turn sampler. The Journal of Machine Learning Research, 15, 1593–1623.

[r23] Kenny, D. A. , & La Voie, L. (1984). The social relations model. Advances in Experimental Social Psychology, 18, 141–182.

[r24] Kim, J. , & Wilson, M. (2020). Polytomous item explanatory item response theory models. Educational and Psychological Measurement, 80(4), 726–755. 32616956 10.1177/0013164419892667 PMC7307487

[r25] Lewandowski, D. , Kurowicka, D. , & Joe, H. (2009). Generating random correlation matrices based on vines and extended onion method. Journal of Multivariate Analysis, 100(9), 1989–2001.

[r26] Linacre, J. M. (1989). Many-faceted Rasch measurement [Unpublished doctoral dissertation]. The University of Chicago.

[r27] Luaces, O. , Díez, J. , & Bahamonde, A. (2018). A peer assessment method to provide feedback, consistent grading and reduce students’ burden in massive teaching settings. Computers & Education, 126, 283–295.

[r28] Ma, Y. , & Genton, M. G. (2010). Explicit estimating equations for semiparametric generalized linear latent variable models. Journal of the Royal Statistical Society: Series B (Statistical Methodology), 72(4), 475–495.

[r29] Martinková, P. , Bartoš, F. , & Brabec, M. (2023). Assessing inter-rater reliability with heterogeneous variance components models: Flexible approach accounting for contextual variables. Journal of Educational and Behavioral Statistics, 48(3), 349–383.

[r30] Masters, G. N. (1982). A Rasch model for partial credit scoring. Psychometrika, 47(2), 149–174.

[r31] Molenaar, D. , Uluman, M. , Tavşanc, E. , & De Boeck, P. (2021). The hierarchical rater thresholds model for multiple raters and multiple items. Open Education Studies, 3, 33–48.

[r32] Namanloo, A. A. , Thorpe, J. , & Salehi-Abari, A. (2022). Improving peer assessment with graph neural networks. In A. Mitrovic & N. Bosch (Eds.), Proceedings of the 15th international conference on educational data mining (pp. 325–332). International Educational Data Mining Society.

[r33] Nemeth, C. , & Fearnhead, P. (2021). Stochastic gradient Markov chain Monte Carlo. Journal of the American Statistical Association, 116(533), 433–450.

[r34] Nestler, S. (2016). Restricted maximum likelihood estimation for parameters of the social relations model. Psychometrika, 81(4), 1098–1117. 26272179 10.1007/s11336-015-9474-9

[r35] Nestler, S. , Geukes, K. , Hutteman, R. , & Back, M. D. (2017). Tackling longitudinal round-robin data: A social relations growth model. Psychometrika, 82(4), 1162–1181. 27924408 10.1007/s11336-016-9546-5

[r36] Nestler, S. , Lüdtke, O. , & Robitzsch, A. (2020). Maximum likelihood estimation of a social relations structural equation model. Psychometrika, 85(4), 870–889. 33094388 10.1007/s11336-020-09728-z PMC8502151

[r37] Nieto, R. , & Casabianca, J. M. (2019). Accounting for rater effects with the hierarchical rater model framework when scoring simple structured constructed response tests. Journal of Educational Measurement, 56(3), 547–581.

[r38] Panadero, E. , & Alqassab, M. (2019). An empirical review of anonymity effects in peer assessment, peer feedback, peer review, peer evaluation and peer grading. Assessment & Evaluation in Higher Education, 44(8), 1253–1278.

[r39] Papaspiliopoulos, O. , Roberts, G. , & Sköld, M. (2007). A general framework for the parametrization of hierarchical models. Statistical Sciences, 22(1), 59–73.

[r40] Patchan, M. M. , Schunn, C. D. , & Correnti, R. J. (2016). The nature of feedback: How peer feedback features affect students’ implementation rate and quality of revisions. Journal of Educational Psychology, 108(8), 1098.

[r41] Patz, R. J. , Junker, B. W. , Johnson, M. S. , & Mariano, L. T. (2002). The hierarchical rater model for rated test items and its application to large-scale educational assessment data. Journal of Educational and Behavioral Statistics, 27(4), 341–384.

[r42] Piech, C. , Huang, J. , Chen, Z. , Do, C. , Ng, A. , & Koller, D. (2013). Tuned models of peer assessment in MOOCs. In S. K. D’Mello , R. A. Calvo , & A. Olney (Eds.), Proceedings of the 6th international conference on educational data mining (pp. 153–160). International Educational Data Mining Society.

[r43] Rasbash, J. , & Goldstein, H. (1994). Efficient analysis of mixed hierarchical and cross-classified random structures using a multilevel model. Journal of Educational and Behavioral Statistics, 19(4), 337–350.

[r44] Raudenbush, S. W. (1993). A crossed random effects model for unbalanced data with applications in cross-sectional and longitudinal research. Journal of Educational Statistics, 18(4), 321–349.

[r45] Reily, K. , Finnerty, P. , & Terveen, L. (2009). Two peers are better than one: Aggregating peer reviews for computing assignments is surprisingly accurate. In S. Teasley , E. Havn , W. Prinz , & W. Lutters (Eds.), Proceedings of the 2009 ACM international conference on supporting group work (pp. 115–124). Association for Computing Machinery.

[r46] Sajjadi, M. S. M. , Alamgir, M. , & Luxburg, U. (2016). Peer grading in a course on algorithms and data structures: Machine learning algorithms do not improve over simple baselines. In Proceedings of the third ACM conference on learning at scale (pp. 369–378).

[r47] Samejima, F. (1969). Estimation of latent ability using a response pattern of graded scores. Psychometrika, 34(Suppl 1), 1–97.

[r48] Sanchez, C. , Atkinson, K. , Koenka, A. , Moshontz, H. , & Cooper, H. (2017). Self-grading and peer-grading for formative and summative assessments in 3rd through 12th grade classrooms: A meta-analysis. Journal of Educational Psychology, 109(8), 1049–1066.

[r49] Stan Development Team. (2023). RStan: The R interface to Stan. (R package version 2.32.3).

[r50] Uto, M. (2021). A multidimensional generalized many-facet Rasch model for rubric-based performance assessment. Behaviormetrika, 48, 469–496.

[r51] Uto, M. , & Ueno, M. (2020). A generalized many-facet Rasch model and its Bayesian estimation using hamiltonian Monte Carlo. Behaviormetrika, 47, 1–28.

[r52] Vehtari, A. , Gelman, A. , & Gabry, J. (2017). Practical Bayesian model evaluation using leave-one-out cross-validation and WAIC. Statistics and Computing, 27, 1413–1432.

[r53] Wang, W.-C. , Su, C.-M. , & Qiu, X.-L. (2014). Item response models for local dependence among multiple ratings. Journal of Educational Measurement, 51(3), 260–280.

[r54] Warner, R. M. , Kenny, D. A. , & Stoto, M. (1979). A new round robin analysis of variance for social interaction data. Journal of Personality and Social Psychology, 37(10), 1742.

[r55] Wilson, M. , & De Boeck, P. (2004). Descriptive and explanatory item response models. In Explanatory item response models: A generalized linear and nonlinear approach (pp. 43–74). Springer.

[r56] Wilson, M. , & Hoskens, M. (2001). The rater bundle model. Journal of Educational and Behavioral Statistics, 26(3), 283–306.

[r57] Xiao, Y. , Zingle, G. , Jia, Q. , Shah, H. R. , Zhang, Y. , Li, T. , … Gehringer, E. F. (2020). Detecting problem statements in peer assessments. In A. N. Rafferty , J. Whitehill , C. Romero , & V. Cavalli-Sforza (Eds.), Proceedings of the 13th international conference on educational data mining (pp. 704–709). International Educational Data Mining Society.

[r58] Xu, J. , Li, Q. , Liu, J. , Lv, P. , & Yu, G. (2021). Leveraging cognitive diagnosis to improve peer assessment in moocs. IEEE Access, 9, 50466–50484.

[r59] Yin, S. , Chen, F. , & Chang, H. (2022). Assessment as learning: How does peer assessment function in students’ learning? Frontiers in Psychology, 13, 912568. 35832911 10.3389/fpsyg.2022.912568 PMC9271947

[r60] Zong, Z. , Schunn, C. D. , & Wang, Y. (2021). What aspects of online peer feedback robustly predict growth in students’ task performance? Computers in Human Behavior, 124, 106924.

