# Supplementary material for: Unfolding the Network of Peer Grades: A Latent Variable Approach
Source: Psychometrika. 2025 Jun 16;90(3):1153–74. doi: 10.1017/psy.2025.10021 (PMC12483701; doi:10.1017/psy.2025.10021)
Supplement: Mignemi et al. supplementary material [file S0033312325100215sup001.pdf]

Supplementary Materials for: Unfolding the Network of Peer Grades: A Latent Variable Approach

1 Mild misspecified scenario

We conducted a small simulation study to evaluate the model's performance under mild misspecification. Specifically, we investigated the accuracy of our proposal estimates when student-specific features do not follow a multivariate normal distribution. We generated $R = 10$ independent datasets from the Main Model, as in the prior sensitivity analysis in Appendix C, but with the student-specific features $(\alpha_i, \beta_i, log(\eta_i^2), log(\phi_i^2))$ independently drawn from a multivariate skew t-distribution $ST(\boldsymbol{\mu}, \boldsymbol{\sigma}, \boldsymbol{\gamma}, \nu)$ where the location vector $\boldsymbol{\mu}$ and the covariance matrix $\boldsymbol{\Sigma}$ are the same as in the prior sensitivity analysis, and where $\boldsymbol{\gamma} = \mathbf{2}_4$ is a four-dimensional vector and $\nu = 20$ are the degrees of freedom of the distribution.
We used the root mean square error (RMSE) and the mean absolute error (MAE) to measure the accuracy of the model's estimates. The priors and the model's computational details are identical to the first scenario in Appendix C.

As Table 1 shows, the estimates' accuracy is reasonable and comparable to those provided by the proposal when the model is correctly specified.

Parameter	RMSE	MAE
δ_1	0.625	0.608
δ_2	0.635	0.614
δ_3	0.690	0.669
δ_4	0.681	0.664
μ_3	0.139	0.120
μ_4	0.067	0.057
σ_1	0.113	0.088
σ_2	0.091	0.081
σ_3	0.119	0.114
σ_4	0.077	0.064
ω_{12}	0.439	0.168
ω_{13}	0.029	0.022
ω_{14}	0.032	0.023
ω_{23}	0.020	0.015
ω_{24}	0.055	0.039
ω_{34}	0.004	0.003
True score	0.706	0.498

Table 1: Root Mean Square Error (RMSE) and Mean Absolute Error (MAE) related to students' true scores and structural parameters across 10 independent data sets.

2 Score reliability and multiple grades

We provide some examples of the role of grader reliability on the estimates of students' true scores. More specifically, does the accuracy decrease when students are unreliable as graders? Third, we want to know whether a larger number of graders per student might mitigate reliability concerns. Namely, to what extent does a larger number of repeated measures of the same student's coursework improve the estimated grades' accuracy?

We sampled eight independent datasets from the main model, as Appendix C shows, and eight from the reduced version for only one assignment. Datasets have different sample sizes $N = \{50, 200\}$, number of graders per students' assignment $|S_{it}| = \{3, 6\}$ and grader mean reliability $\mu_4 = \{1, -1\}$. We note that $\mu_4 = 1$ implies larger graders' variances $\{\sigma\}_1^N$ and a consequence lower graders reliability levels. On the contrary, $\mu = -1$ implies smaller values for $\{\sigma\}_1^N$ and higher reliability levels.

2.1 Results

The mean square error (MSE) and the person correlation coefficient r between the true score and the estimated ones are reported in Table 2 and 3 for the multiple and single assignment datasets, respectively, along with the respective scatter plots. The grades provided by our proposal are, on average, more accurate for higher graders' reliability

levels. A larger number of graders per student assignment increases the accuracy of grades estimates, and specifically, this improvement is greater when graders are poorly reliable.

3 Additional results from real data

We present additional results regarding the multiple assignment real dataset analyzed in Section 3.1. Specifically, we report the scatter plots and the Pearson correlation coefficients between the posterior means of the students' features in Figure 1 below. The density of these point estimates of each student-specific latent variable is represented along the main diagonal. We observe that the posterior mean of the student's average ability α is negatively correlated with their systematic bias τ. Students with high average proficiency levels have a lower systematic bias; they tend to give smaller grades to their peers' works. On the contrary, low average proficiency levels are associated with larger systematic biases; these students tend to provide larger grades to their peers' works.

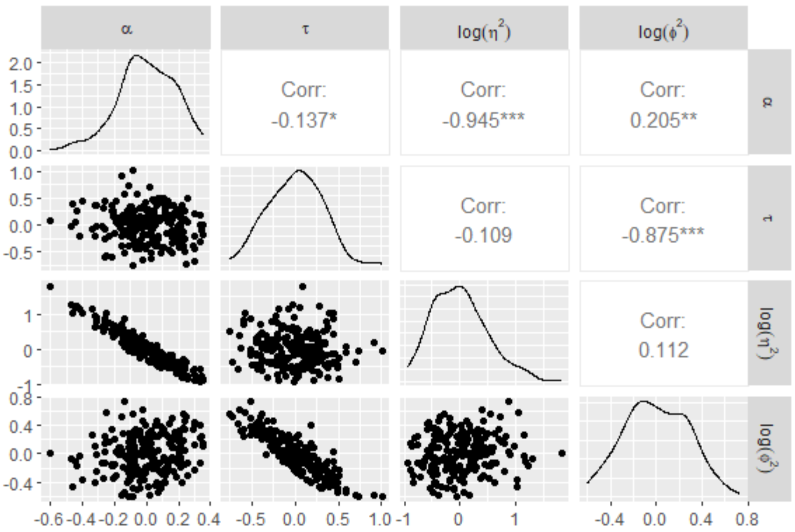

Figure 1: Results about the posterior mean estimates of the student-specific latent variables, including their density plots, pairwise scatter plots, and Pearson correlations between latent variables.

	$\mu_4 = 1$	$\mu_4 = -1$
N = 50		
$\lvert S_{it} \rvert = 3$	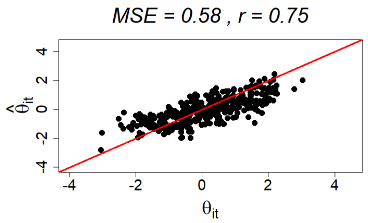	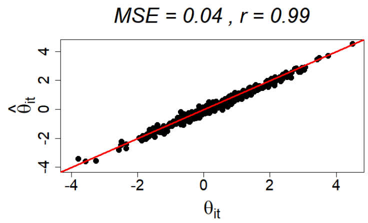
$\lvert S_{it} \rvert = 6$	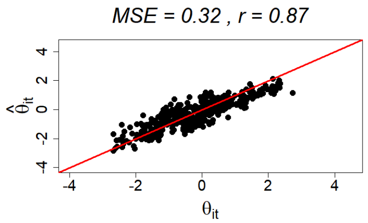	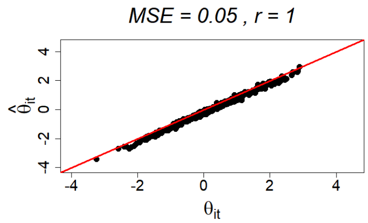
N = 200		
$\lvert S_{it} \rvert = 3$	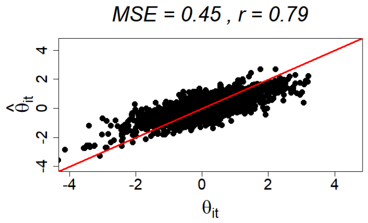	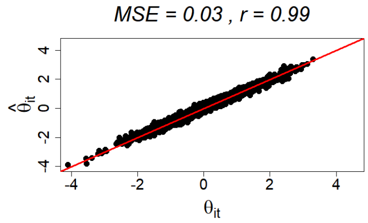
$\lvert S_{it} \rvert = 6$	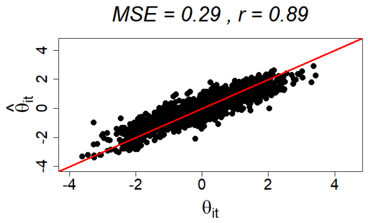	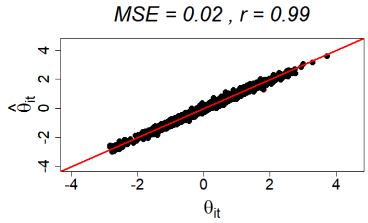

Table 2: For each simulation scenario the true value of θ_{it} against the posterior mean estimate $\hat{\theta}_{it}$ is plotted. A 45-degree line is plotted to highlight possible under- or over-estimate trends. Here μ_4 is the mean grader reliability, smaller values imply higher reliable graders; $|S_{it}|$ is the number of grader per coursework. The sample size, i.e. the total number of students, is indicated by N.

	$\mu_4 = 1$	$\mu_4 = -1$
N = 50		
$\lvert S_{it} \rvert = 3$	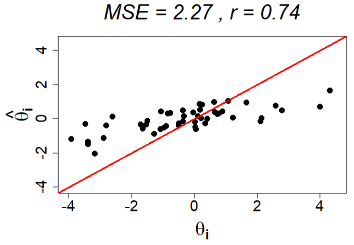	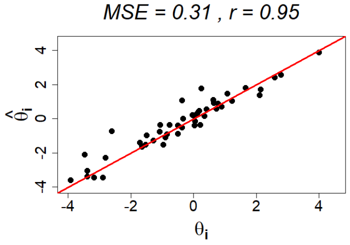
$\lvert S_{it} \rvert = 6$	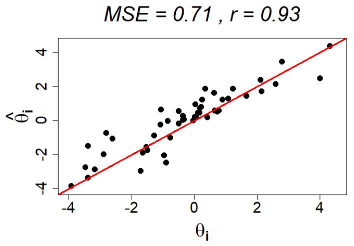	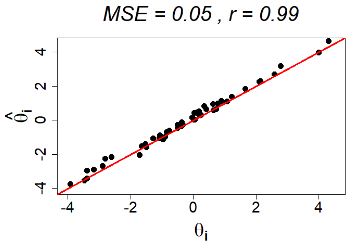
N = 200		
$\lvert S_{it} \rvert = 3$	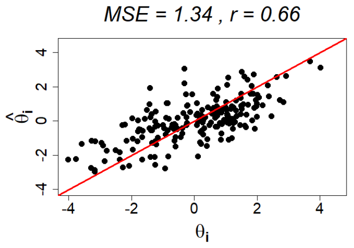	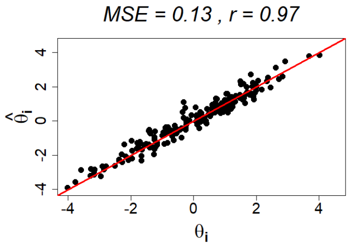
$\lvert S_{it} \rvert = 6$	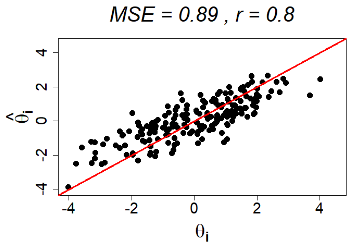	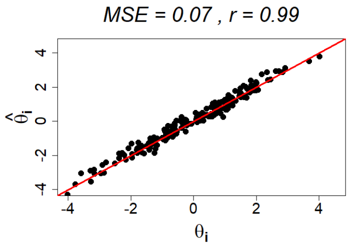

Table 3: For each simulation scenario the true value of θ_{it} against the posterior mean estimate $\hat{\theta}_{it}$ is plotted. A 45-degree line is plotted to highlight possible under- or over-estimate trends. Here μ_4 is the mean grader reliability, smaller values imply higher reliable graders; $|S_{it}|$ is the number of grader per coursework. The sample size, i.e. the total number of students, is indicated by N.
